# Evaluation of phosphopeptide enrichment strategies for quantitative TMT analysis of complex network dynamics in cancer-associated cell signalling

**DOI:** 10.1016/j.euprot.2015.01.002

**Published:** 2015-02-07

**Authors:** Benedetta Lombardi, Nigel Rendell, Mina Edwards, Matilda Katan, Jasminka Godovac Zimmermann

**Affiliations:** a Proteomics and Molecular Cell Dynamics, Center for Nephrology, School of Life and Medical Sciences, University College London, Royal Free Campus, Rowland Hill Street, London NW3 2PF, United Kingdom; b Wolfson Drug Discovery Unit, Centre for Amyloidosis and Acute Phase Proteins, Division of Medicine, University College London, London NW3 2PF, United Kingdom; c Institute of Structural and Molecular Biology, Division of Biosciences, University College London, Gower Street, London WC1E 6BT, United Kingdom

**Keywords:** Peptides immunoprecipitation, Phosphopeptides enrichment, TMT, Titanium dioxide

## Abstract

Defining alterations in signalling pathways in normal and malignant cells is becoming a major field in proteomics. A number of different approaches have been established to isolate, identify and quantify phosphorylated proteins and peptides. In the current report, a comparison between SCX prefractionation *versus* an antibody based approach, both coupled to TiO_2_ enrichment and applied to TMT labelled cellular lysates, is described. The antibody strategy was more complete for enriching phosphopeptides and allowed the identification of a large set of proteins known to be phosphorylated (715 protein groups) with a minimum number of not previously known phosphorylated proteins (2).

## Highlights

• An overview is presented to highlight critical steps of quantitative phosphoproteomics.
• Mass spectrometry limitations in phosphpo-peptides identification.
• A comparison of two experimental workflows (SCX and antibody based) to enrich phosphopeptides shows that the two methods detect different phosphorylation sites.

## Graphical abstract

**Figure:**
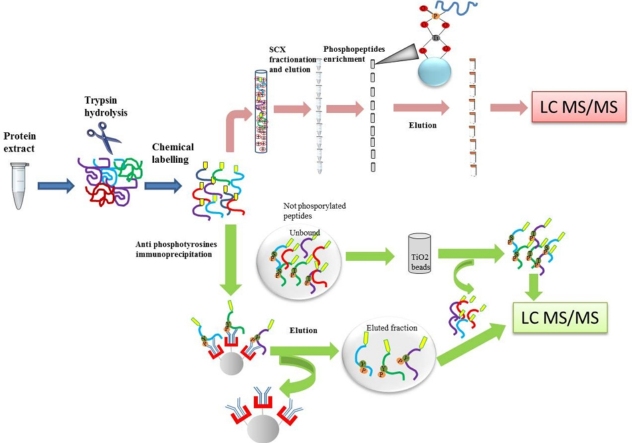

Many biological functions of living cells are regulated by phosphorylation/dephosphorylation of proteins. These reactions are crucial in controlling key aspects of protein function, including interactions in signalling pathways, activation or inactivation and subcellular localization. Monitoring the status of phosphorylation of proteomes became a major goal in basic as well as in applied research because it offers a unique tool to unravel signalling pathways and to identify crucial nodes that can be altered during disease development. Non MS-based strategies, mainly relying on the use of protein arrays, have been used in the past to identify kinases substrates [1] or to unravel regulatory mechanisms of specific signalling pathways [2], but these approaches lack the possibility to precisely map the phosphorylation site, discover novel sites, or differentiate between different phospho-sites within the same protein. For these reasons, plus the need for high throughput studies, MS-based strategies are becoming the methods of choice in the field.

Although MS-based strategies offer the best tool to precisely identify and map phospho-sites, two major issues complicate the detection of phosphorylations: (i) low stoichiometric abundance in the proteome, and (ii) low efficiency of MS fragmentation and/or loss of the phosphoric group. At a given time, only a small proportion of the proteins present in a proteome are phosphorylated and the phosphorylation status of the same protein can vary within the same protein lysates [3]. Hence, enrichment strategies to specifically isolate phosphoproteins or phosphopeptides must be undertaken before proceeding with mass spectrometry analysis. Furthermore, identifying and mapping phospho-sites in a sample requires special care in setting up an appropriate MS–MS methodology. The CID approach, which is often used for peptide sequencing in shotgun proteomic experiments, is not necessarily the method of choice for maximal identification of phosphorylation sites. Because of the neutral loss occurring for phosphoserine peptides and, to a lesser extent, for phosphothreonine peptides, sequencing information is often lost following the fragmentation of these peptides by CID. Many different fragmentation approaches have been tested and suggested over recent years to address this issue, including MSA [4], HCD [5] and ETD [6], but a full consensus has not been achieved for any of them. It seems that the success of one approach *versus* another depends on the sample complexity, LC-setup, and specific MS settings [7]. Phosphotyrosine peptides are even more difficult to identify, both because of the lower level of tyrosine phosphorylation compared to serine and threonine [8] and because of the dynamic nature of tyrosine phosphorylation.

Further constraints may arise from the nature of the biological samples. Our target is the telomerase immortalized human urothelial cell-line (TERT-NHUC) stably transfected with FGFR IIIb or with a fusion form of the same receptor (RT112FUS) [9]. To prevent malignant transformation, it is necessary to limit the number of passages for this cellular system. This precludes metabolic labelling methods such as SILAC and dictates the use of *in vitro* chemical labelling. This, in turn, implies enrichment of peptides rather than proteins because of the loss in trypsin digestion efficiency after chemical labelling (due to lysine modification with reporter tags) and this is applicable and cost effective for a limited amount of sample (≤1 mg). The isobaric mass tag reagents iTRAQ or TMT are the most commonly used compounds to label peptides *in vitro*. They both are based on a N-hydroxysuccinimide (NHS) chemistry which allows them to react with every N-terminus and ɛ-amine group of lysines, assuring that every tryptic peptide could potentially be labelled. The main difference between iTRAQ and TMT is the delta mass added to each labelled peptide and the molecular weight of the reporter ion generated after fragmentantion, which gives the relative quantitation of each peptide. In terms of number of samples that can be simultaneously quantified, iTRAQ allows up to 8 independent analyses (iTRAQ 8-plex) while TMT up to 10 (TMT 10-plex). These two reagents have been compared to assess if one outperforms the other in terms of numbers of identified peptides/proteins and quantitation accuracy [10], [11], but a clear conclusion has not been determined. It seems that different factors (*e.g.* scoring factors, search algorithms, instrument used) may affect the final result [12], [13] and choosing one reagent over the other can just depend on the number of independent samples handled. In this study, the TMT 6-plex has been chosen.

We present in this report a comparison of two quantitative phosphoproteomic workflows, both suitable to quantitatively evaluate network dynamics in cells. Since it is known that FGFR activation triggers a series of events involving tyrosine phosphorylation [14], particular attention was paid to this specific modification. Samples were subjected to two different workflows: (i) Strong Cation Exchange (SCX) chromatography coupled to titanium dioxide enrichment (SCX + TiO_2_), and (ii) phosphotyrosine immunoprecipitation coupled to phosphopeptide enrichment of the unbound fraction after IP with titanium dioxide (α-pYs IP+ Unbound TiO_2_) (Fig. 1). To set up the protocol, the A431 human epithelial carcinoma cell line stimulated with EGF1 has been used. It is a well-known model to study cancer associated signalling pathways and phosphorylation events [15], [16].

**Fig. 1:**
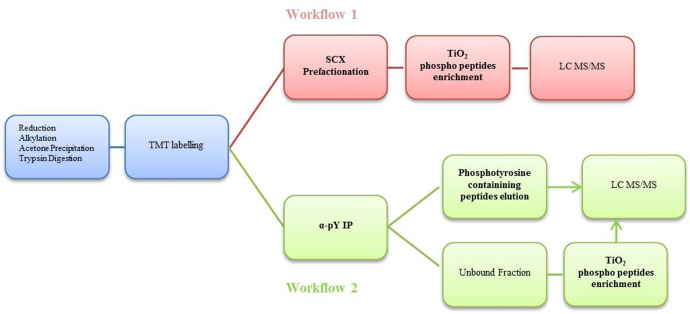
Overview of the experimental procedures. Schematic representation of workflow 1 (SCX prefractionation + titanium dioxide enrichment) and Workflow 2 (IP + titanium dioxide enrichment). Underlined in bold are crucial nodes of each workflow. SCX: Strong Cation Exchange; TiO_2_: titanium dioxide; α-pY IP: anti-phosphotyrosine Immuno precipitation.

All samples were separated by reverse phase HPLC over a 130 min gradient before MS/MS (Buffer A: 0.1% formic acid in water, Buffer B: 0.1% formic acid in acetonitrile; 0–1 min, 1% B; 1–90 min, 50% B; 90–91 min, 85% B; 91–111 min, 85% B; 111–112 min 85–1% B; 112–130 min, 1% B).

Cells were lysed by adding RIPA buffer containing protease and phosphatase inhibitors and protein concentration was measured using the DC protein concentration kit (Biorad). 800 μg of proteins were reduced, alkylated and precipitated overnight at 4 °C by adding 6 volumes (v/v) of cold acetone. After centrifugation (15 min at 10,000 × *g*), the resulting pellet was resuspended in 0.1 M of TEAB (triethyl ammonium bicarbonate), trypsin was added at 1:100 ratio and digestion was carried out for 16 h at 37 °C. The labelling reaction was conducted using TMT reagents (Thermo Scientific), adding 1.6 mg of labelling reagents and then following manufacturer's instructions. Labelling efficiency was evaluated by analysing 1/100 of the sample with a LTQ-Velos mass spectrometer (Thermo Fisher Scientific) coupled to a Nanoacquity UPLC (Waters, U.K.). Labelling efficiency was evaluated by counting the number of peptides identified as labelled over the number of all identified peptides with a set F.D.R. of 0.05 and it was estimated to be 97% (data not shown). Since labelling follows extraction of the phosphopeptides, after ensuring a suitable labelling procedure was available, the comparison between the enrichment workflows described in the following was conducted on unlabelled samples. The LTQ-Velos mass spectrometer was set up as described by Johnson et al. [17] basically the 10 most intense peaks detected in each cycle have been fragmented by CID and HCD. The combination of these 2 approaches might increase the sensitivity of phosphopeptide detection [18] and both fragmentation approaches are known to be compatible with the TMT quantitative approach [19], [20].

After trypsin digestion, samples were subjected either to workflow 1 (SCX + TiO_2_) or to workflow 2 (α-pYs IP + Unbound TiO_2_). For workflow 1, the SCX procedure was performed accordingly to Villen et al. [21] with the difference that the fractionation was done using Mini Ion Exchange Spin Columns (Thermo Scientific) that are more suitable for the amount of material available. Fractions were collected at: 5, 10, 15, 20, 25% of Buffer B (30% CH_3_CN, 7 mM KH_2_PO_4_ pH = 2.7, 350 mM KCl) in Buffer A (30% CH_3_CN, 7 mM KH_2_PO_4_ pH = 2.7); 100% of Buffer B, 100% of Buffer C (500 mM NaCl, 50 mM KH_2_PO_4_ pH = 7). In addition, the column flow through after sample loading and the first column wash with 100% of Buffer A were saved and subjected to further phosphopeptide enrichment. A total of 9 fractions were collected, dried and then subjected to the phosphopeptide enrichment step using the TiO_2_ phosphopeptide Enrichment kit (Thermo Scientific) followed by a clean-up step with graphite columns (Thermo Scientific) according to manufacturer's instructions. The resulting samples were dried, resuspended in 0.1% of formic acid and analyzed with the LC–MS/MS system.

Workflow 2 was designed to couple a procedure specifically aimed at enrichment of phosphotyrosine peptides with the TiO_2_ enrichment strategy. Phosphotyrosine immunoprecipitation was conducted by incubating 800 μg of tryptic peptides resuspended in IP buffer (100 mM Tris, 1% Nonidet P-40, pH 7.4) with Protein G agarose beads coupled to a mixture of 3 different anti-phosphotyrosine antibodies (pY100, 4G10 and PT66) following the procedure described by Johnson et al. [17]. After overnight incubation with antibody beads, the sample was briefly spun down and the supernatant (unbound fraction) was saved and dried for phosphopeptide enrichment with TiO_2_ as described above. Phosphotyrosine peptides were eluted by twice adding to the beads 70 μl of 0.1 M Glycine, pH 2. Both samples (the eluted fraction after IP and the TiO_2_ enriched sample) were dried, resuspended in 0.1% formic acid and analyzed by LC–MS/MS.

For data analysis, Proteome Discoverer 1.3 was used, searching against the UniProt human database with the Mascot search engine. Up to 2 trypsin missed cleavages were allowed, carbamidomethylation was set as a fixed modification with methionine oxidation and phosphorylation of serine, threonine and tyrosine as variable. Mass tolerance was set to 10 ppm for the precursors and to 0.8 Da for the fragments. The chosen false discovery rate was 0.05, with subsequent manual validation of the peptides containing pY [13], [22], [23].

Workflow 1 led to the identification of 3121 peptides, corresponding to 1183 protein groups, while workflow 2 identified 1720 peptides associated to 717 protein groups. However, workflow 2 was more specific than workflow 1 for identification of phosphopeptides, with a percentage of 43% *versus* 23% (Fig. 2a). If only peptides identified from the “Unbound-TiO_2_” fraction of workflow 2 are taken into account, then the specificity of this protocol increases to 83% (Fig. 2b), clearly showing that the phosphopeptide identification rate from the IP procedure was less satisfactory. Focusing on workflow 1 results, we note that each SCX fraction (except for fraction 7), contributes to the structure of the final dataset of identified phosphopeptides by including peptides uniquely present in that fraction (Fig. 2b). This shows that every fraction is enriching a specific, unique pool of phosphopeptides.

**Fig. 2:**
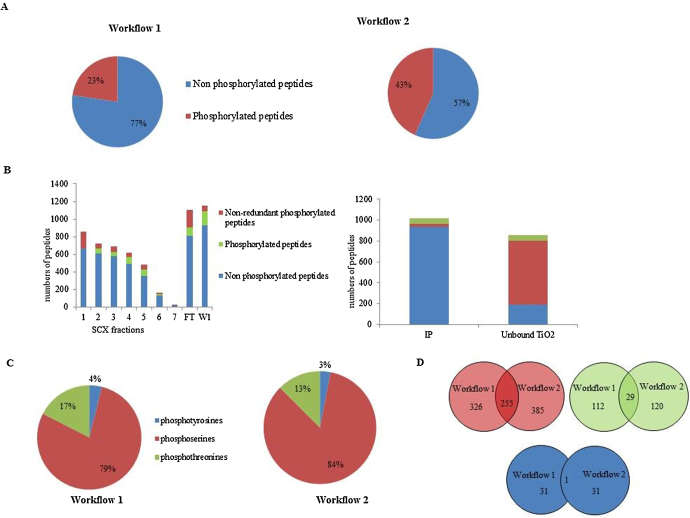
Schematic summary of the two datasets. (a) Pie charts showing the percentage of non phosphorylated and phosphorylated peptides recovered using the two workflows. (b) Histograms showing the distribution of non phosphorylated peptides, non-redundant phosphorylated peptides and phosphorylated peptides identified in each fraction of the two workflows. (c) Pie charts showing the percentage of phosphoserines, phosphothreonines and phosphotyrosines sites identified in the two workflows. (d) Venn diagrams showing the number of non-redundant pS, pT and pY phosphopeptides detected with workflow 1, workflow 2 or both workflows.

Looking at the number of serines, threonines and tyrosines identified as phosphorylated, the percentage of each of them reflects the expected distribution within proteomes, with the highest percentage given by phosphoserines (79–84% in our datasets) and the lowest given by phosphotyrosines [8]. We note that, compared to the phosphosite distribution evaluated in a previous work, but with a different cell line (HeLa cells) [24], both datasets shown here, have a higher proportion of identified phospho-tyrosine sites (4% in workflow 1 and 3% in workflow 2), thus emphasizing the crucial role of phosphotyrosines in EGF pathways. This result is promising for workflow 1, where no specific step attempted to enrich phosphotyrosines, while it is less satisfactory for workflow 2, where a higher proportion of phosphotyrosines was anticipated. Conversely, phosphotyrosines were not detected with TiO_2_ extraction alone (no immunoprecipitation), *i.e.* workflow 2 enriched a set of phosphotyrosines that were only partially detected with workflow 1.

At the level of identified protein groups, using workflow 2, 717 proteins were identified, of which 715 are known phosphoproteins (based on the PhosphoSitePlus database [25]) while with workflow 1, 824 out of 1183 are known phospho-proteins (Fig. 2C). Focusing on those proteins identified based on peptides enriched after immunoprecipitation, 335 (out of 386) are known to have phosphotyrosine sites (Supplementary Table 1). Notably, the pS, pT and pY peptides identified with workflows 1 and 2 were only partially common (Fig. 2D). These observations suggest that three sources may constrain the identification of phosphorylation sites: a lack of specificity in successfully enriching phosphorylated peptides, loss of the phosphate group during sample preparation and fractionation, and unsuccessful MS/MS analysis.

Supplementary Table 1 related to this article can be found, in the online version, at doi:10.1016/j.euprot.2015.01.002.

Supplementary Table 1 Table listing all the proteins identified in both workflows, with the indication of known phosphoproteins, the presence of known phosphotyrosine sites within each protein, the percentage of sequence coverage (ΣCoverage), the indication of unique identified peptides (Σ# Unique Peptides) and total number of identified peptides (Σ# Peptides) associated to each protein.

To further explore this hypothesis, both datasets were mined using the PhosphoSitePlus database to look for the possible presence of (i) certain sources of contamination and, (ii) for sequences known to be phosphorylated, but identified as not phosphorylated in the current analysis. Contaminating peptides (i) can be grouped in 2 categories: peptides not having serines, threonines or tyrosines in their sequences (379 in workflow 1 and 104 in workflow 2) and peptides containing these amino acids, but having a sequence not previously reported to be phosphorylated (525 in workflow 1 and 311 in workflow 2). The first group virtually certainly represents contamination because there is no available evidence (at the sequence, the database and the dataset level), at the current state-of-the-art, that these sequences could have a phosphorylated site. The second level of the analysis (ii) unravelled those peptides that present evidence of possible phosphorylation (because they have already been described as phosphorylated and they have been recovered after a specific phosphopeptide enrichment procedure). This group might still contain contaminant peptides (*i.e.* peptides known to be phosphorylated, but in cell lines different from the one under investigation), but considering that a specific enrichment strategy has been applied to all samples to isolate phosphopeptides, it is more likely that the phosphosite was not identified due to the loss of the phosphoric group during the MS/MS analysis or to an unsuccessful MS/MS analysis. Indications that this can be a substantial problem are apparent in the 56 peptides that were detected in both workflows, but were verified as phosphorylated in only one of the workflows. This second group (ii) represents 48% of the non-redundant identified sequences in workflow 1 and 26% in workflow 2. In summary, as shown in Table 1, the final number of non-redundant, identified sequences, having no possibility to contain pS, pT or pY, is 904 for workflow 1 (30% of the entire dataset) and 415 (25%) for workflow 2. Taken together, these data suggest that workflow 2 has a slightly higher level of specificity in enriching peptides from known phosphorylated proteins and the resulting phosphopeptide dataset contains approximately 75% peptides known to be phosphorylated.

**Table 1:** The potential number of contaminant sequences identified in both workflows. S: Serine; T: Threonine; Y: Tyrosine.

Sequences	Workflow 1	Workflow 2
Total number of identified peptides	3121	1720
Total number of non-redundant identified sequences	2924	1626
Total number of peptides identified as not phosphorylated	2413	974
Total non-redundant sequences identified as not phosphorylated	2307	941
Number of peptides, identified as not phosphorylated and not containing S,T and Y	401	107
Number of non-redundant sequences, identified as not phosphorylated and not containing S,T and Y	379	104
Number of peptides, identified as not phosphorylated, but containing S,T,Y	2012	867
Number of non-redundant sequences, identified as not phosphorylated, and containing S,T,Y	1929	838
Number of non-redundant sequences, identified as not phosphorylated, containing S,T,Y and known to be potentially phoshorylated	1405	526
**Total number of potential contaminant sequences**	**904**	**415**
Percentage of potential contaminant sequences over the number of non-redundant sequences identified as not phosphorylated	39	44
**Percentage of potential contaminant sequences over the number of total non-redundant sequences identified**	**31**	**26**

The set of identified phosphotyrosine sites may still be incomplete. The type of identified proteins suggest these sites may be phosphorylated in the sample, but the subsequent sample preparation, fractionation and MS mapping compromise the positive confirmation of phosphorylation at these sites. This could partially depend on the low abundance of phosphotyrosines and partially on the presence of detergents, coming from the sample preparation, which can affect the overall ionization efficiency and subsequent MS/MS analysis [26]. It is likely that removing interfering agents after the immunoprecipitation would improve spectral quality and, therefore, phosphotyrosine sites identification [27].

In conclusion, to define the most productive approach to a quantitative study of phospho-sites present in a sample-limited cellular system, a comparison between a phosphopeptide enrichment strategy coupling SCX fractionation with TiO_2_ enrichment and an approach combining phosphotyrosine enrichment and TiO_2_ enrichment of the remaining phosphoserine and phosphothreonine containing peptides, has been conducted. Considering the number of identified phospho-sites as well as the proportion of known phosphopeptides and phosphoproteins finally detected, workflow 2 gave slightly more promising results and may still be substantially improved by removing detergents after the immunoprecipitation. Although the SCX prefactionation coupled to TiO_2_ showed lower specificity, it has the potential to unravel a higher number of peptides, thanks to the lysate pre-fractionation performed before phosphopeptide isolation. Conversely, although it has become a widely used procedure, SCX prefractionation has been reported to have substantial losses of hydrophobic phosphopeptides and poorer specificity than reverse phase separations [28]. Jointly the two workflows positively identified more phosphorylation sites (1302) than either workflow individually. That is, there still seems to be room for improvements in commonly used phosphoproteome protocols, especially in the context of sample-limited analyses, and comprehensive coverage of phosphorylation sites may require use of multiple protocols.

## Transparency document

Transparency document
